# Turkish medical educators’ TPACK components and characters: an analysis within the framework of simulation-based medical education

**DOI:** 10.1186/s12909-019-1664-1

**Published:** 2019-06-25

**Authors:** Engin Karadag

**Affiliations:** Akdeniz University, 07070 Konyaaltı, Antalya Turkey

**Keywords:** Medical education, Simulation-based medical education, TPACK, Educational technology, Teaching/learning strategies

## Abstract

**Background:**

Nowadays, a comprehensive approach is needed to describe the current status of the technology integration process and the identification of the factors that affect it, because the description and frame of the existing situation will be the starting point in the organization of the roadmap to the realization of an effective integration process. The purpose of this study is to identify the differences in technology integration of the medical educators working in Turkey and analyze these according to various variables on the basis of technological pedagogical content knowledge.

**Method:**

Data used in the study were collected from 301 medical educators using the *TPACK-Practical Scale* and Attitude towards Technology Scale. The data were analyzed using Ward’s minimum variance hierarchical clustering analysis, discriminant function analysis and multinomial logistic regression analysis.

**Results:**

According to the results of the study, medical educators’ technology integration was grouped in the following clusters, according to their TPACK: (*i*) *activity-based,* (*ii*) *student- based* and (*iii*) *topic-based*. It was found that the developed model explains 79*%* of the variance of technology integration. The implementation of simulation-based medical education in medical school and the department where they work affect the clusters to which the medical educators were assigned, whereas the gender variable didn’t have an effect. The findings showed that attitudes towards technology, simulation education and working in the field of basic medical sciences increased medical educators’ activity-based presence.

**Conclusions:**

The review of the clusters and their characteristics showed that there are similarities between the items used in the designing stage of the education programs and the relationships of these items among them. Learner centered approaches are based on the assumption that students are located at the center of the program. In these designs, teaching mostly focuses on the learner, rather than program, learning or administrative body. Individuals and their identities are crucial.

## Background

The acceptance and implementation of a new technology is very similar to the process of accepting an innovation. According to Rogers [1], based on the studies conducted on individuals and communities, the investigation of the adaptation to an innovation and the process of acceptation for different persons are of great importance. Today, where the pace of technologic developments is increasing, many researchers, featuring the technology adaptation of the educators and effective usage of the technological devices are conducted [2–4].

Simulation is a method in which the students gain artificial and virtual experience in an activity which reflects real life circumstances without taking the risk of real life situations [5, 6]. The use of simulation in medicine dates back to the 1950’s. Early medicine simulators were manikins which were named ‘phantom’ in the 16th and 17th centuries. These practices stand out as nonsystematic practices in teaching and examining obstetric skills with the aim of decreasing maternal-infant mortality. The first critical rise in medical simulation came out with Ressusi-Anne which was a result of collaborative work of anesthetists and the industry in twentieth century. This manikin became an example for other models and designs in terms of resuscitation and basic skills education [7, 8]. Current practices of simulation use in medicine include role-playing with simulators (with real or simulated patients), computer-based simulation, simulation software, videos, DVDs or creating virtual reality, computer controlled simulators and interactive patient simulators [5]. Simulation based education is a context which is egalitarian, gives every student an opportunity to learn, uses adult education principles effectively and appeals to different learning styles. In these contexts, interests and needs are defined by the learner and educator, learner experiences are prioritized, learners are given chance to learn by doing and supported with feedbacks. Although it seems to appeal to kinesthetic learners, simulation based medicine education, supported by different theories (behaviorist, cognitivist, constructivist and humanistic), gives chance to individuals to learn in their own style by employing multiple education methods and using various education materials together [6, 9, 10]. Today, thanks to the opportunities provided by technology, different perspectives have emerged about the development of learning environments and technology integration in education. Regarding this rapid change cycle and development of technology, the position of the educators in the process and their activity levels are seen as important issues by the researchers [11].

Nowadays, concrete applications are needed for describing the current status of the technology integration process and the identification of the factors that affect it, because the description and frame of the existing situation will be the starting point in the organization of the roadmap related to its realization. Therefore, the aim of this research is to make a contribution to the description of the current situation of ICT integration in the learning-teaching process from a medical educator dimension and to examine it in terms of the variables that may affect the integration process.

Within the study, the following hypotheses were tested:

***H***_***1***_ Medical educators’ technology integration is clustered according to the TPACK-Practical model.

***H***_***2***_ The clusters formed according to TPACK-Practical skills of the medical educators and the distribution of the medical educators who were working in the schools where simulation-based medical education was implemented are independent.

***H***_***3***_ The clusters formed according to TPACK-Practical skills of the medical educators and the department where they were working are independent.

***H***_***4***_ The clusters formed according to TPACK-Practical skills of the medical educators and their gender distribution are independent.

***H***_***5***_ Medical educators’ seniority, attitude towards technology, the status of simulation-based medical education implementation in their school, the department where they were working and gender variables affect their presence in the clusters formed according to TPACK-Practical skills.

## Related literature

### Technological pedagogical content knowledge (TPACK)

The TPACK model has taken its final shape by integrating the ‘Technology’ dimension with Pedagogical Content Knowledge [PCK], which is a model that features the necessary characteristics the educator should have [12]. The following are components of TPACK; (*i*) technology, which comprises technical knowledge about technological equipment and tools, including tools such as computers, the internet, video, measuring devices, and e-books; (*ii*) pedagogy, which considers teaching methods, strategies, and models and consists of subdomains that include how students learn, how to use classroom management skills, course planning and effective student assessment; and (*iii*) content knowledge, including subject area knowledge, which varies according to grade level and discipline, and all of the theories and ideas of the concepts belonging to a discipline. Pedagogical Content Knowledge [PCK] is the combination of knowledge and pedagogy and involves the presentation of the content area via interactions with pedagogical issues, i.e., the selection of appropriate teaching approaches, methods and techniques. Technological Content Knowledge [TCK] is the combination of technology and content and refers to the use of technology that is more appropriate for representing the subject and content of a particular discipline. Technological Pedagogical Knowledge [TPK] is the combination of technology and pedagogy and considers the effects of technology usage on learning in the teaching process [4, 13–15].

### TPACK-practical model

According to Van Driel Verloop and De Vos [16], educators’ practical knowledge and PCK play an important role in the regulation of the teaching process and in the fulfillment of learning objectives via appropriate teaching strategies. In this context, practical knowledge (teaching experience) with the combined use of content and pedagogy skills is involved in the process as much as PCK. The TPACK model has evolved from different perspectives in the literature and tackle knowledge and skill dimensions independent of teaching experience and performance. From this perspective, the TPACK-Practical model is a model that considers the teaching process as the basis upon which practical knowledge (teaching experience) and TPACK skills work together. The consideration of TPACK and the teaching process together is important in terms of the skills used through the process and the consideration of the interaction between these two processes in addition to providing immediate feedback. Specifically, it should not be ignored that the processes requiring different technologies, such as the recognition of students, planning, design, and evaluation, require different TPACK skills. According to Yeh, Hsu, Wu and Chien [17], in-service educators’ TPACK can be very different from that which pre-service educators develop, because teaching experiences and beliefs can personally vary and intuitively interact. Thus, teaching processes and outcomes are affected by the interaction of possessed knowledge and skills with teaching experience. Jang and Chen [18] stated that the lack of experience and naivety of educators may act as the limiting factor in the use of TPACK skills. Thus, variables, such as the experience and performance of the educator, must be involved in the process. The implementations of TPACK skills in different disciplines are also different. Educators are the key element to introduce ICT into educational practice [19].

**Fig. 1:**
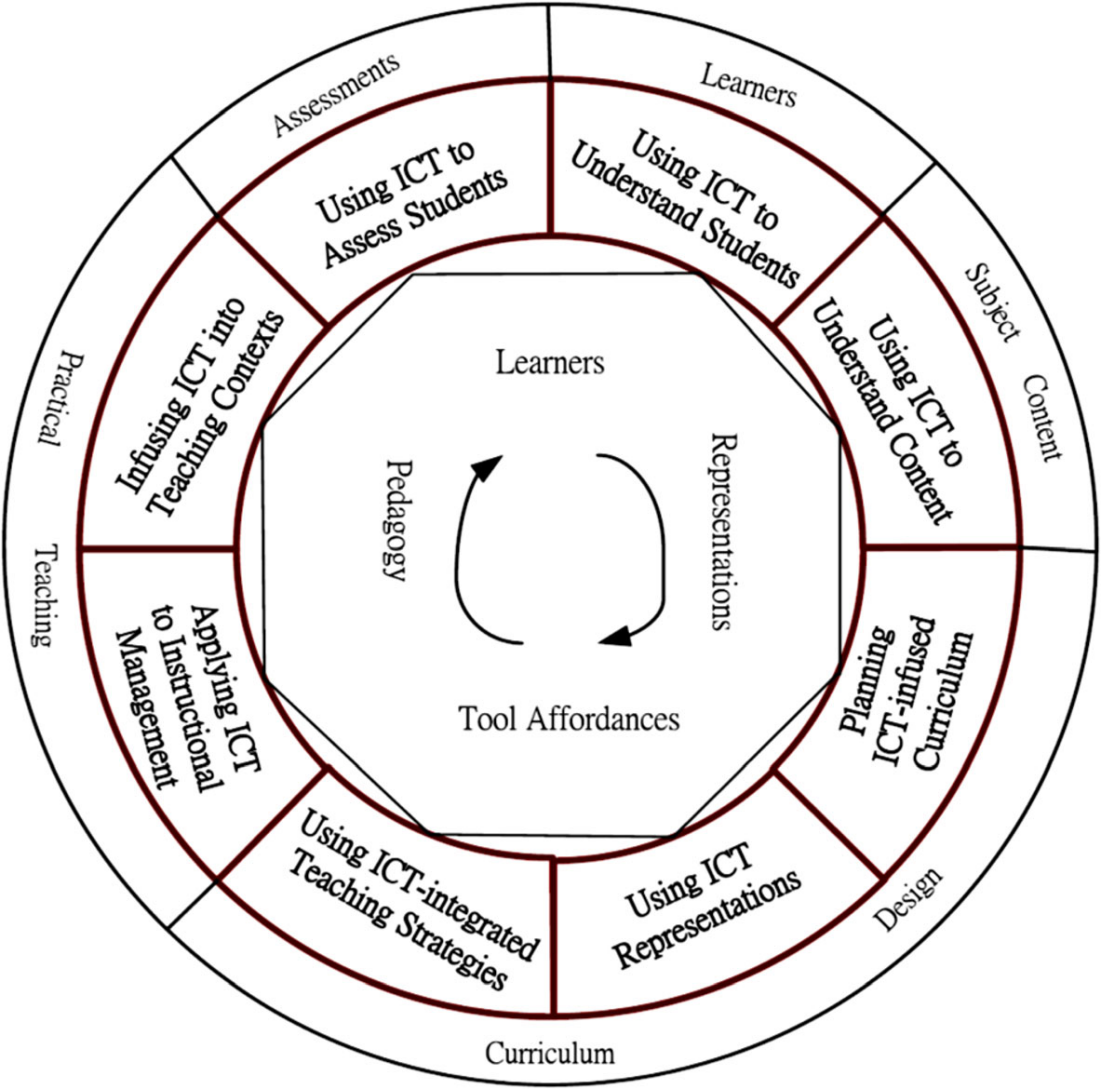
The framework of the TPACK-Practical model

The TPACK-Practical model (see Fig. 1) consists of eight knowledge dimensions from five pedagogical areas. These pedagogical areas include the following: (*i*) learners, (*ii*) subject content, (*iii*) curriculum design, (*iv*) practical teaching, and (*v*) assessment. The knowledge dimensions belonging to these areas are the following: (*i*) using ICT to understand students, (*ii*) using ICT to understand subject content, (*iii*) planning ICT-infused curricula, (*iv*) using ICT representations, (*v*) using ICT- integrated teaching strategies, (*vi*) applying ICT to instructional management, (*vii*) infusing ICT into teaching contexts, and (*viii*) using ICT to assess students [18].

## Methods

### Research design

The research was designed using the correlational method, which is one of the non-experimental quantitative research approaches. The correlational model is the design where the researcher seeks for a relationship between two or more variables that interact with each other. The aim of the study is to reach a decision or a model by analyzing the relationship between variables. Thus, this research is based on the *Explanatory Correlational Design* [20].

### Participants

This study was conducted with 339 medical educators from 14 medical schools that were identified by means of deliberate sampling. The data obtained from 38 medical educators were likely to adversely affect the reliability of the study and were removed before beginning the analysis (these medical educators gave the same scores to all of the items). Therefore, the data obtained from 301 participants were used in the study.

### Data collection tools

#### TPACK-practical scale

The TPACK-Practical Scale items were obtained from the Delphi study conducted by Yeh, Hsu, Wu, Hwang and Lin [19], which was performed in two stages with the participation of 6 researchers and 54 specialists. Regarding the Turkish version of the scale, as a result of item-total (*r* = .44–.65, *p <* .01) and item-rest (*r* = .41–.63, *p <* .01) correlation analysis, it was found that there is a significant relationship for each item in the scale, and the differentiating power was found to be 27*%* and the relationship between lower and upper groups’ averages was found to be significant for all tested items at *p <* .05 level. According to the confirmatory factor analysis conducted, the original structure of the scale was confirmed and, as in the original form, eight knowledge dimensions from five pedagogic domains were revealed. In addition, for internal consistency, Cronbach’s Alpha reliability coefficient of the scale was found to be 0.89.

The content of the scale factors can be described as follows:

(i.) *Learners:* High scores for this factor indicate that the medical educator has gained skills such as recognizing the students using ICT, identifying and resolving the students’ difficulties in the learning process (e.g., misconceptions), identifying the students’ learning styles and following up on their improvement levels.

The following are sample items related to this factor:

1. I know how to use ICT to identify students’ learning difficulties.
2. I am able to use different technology-infused instruction to assist the students with different learning characteristics.

(ii.) *Subject Content:* High scores for this factor indicate that the medical educator has gained skills such as using ICT to learn the content. The following are sample items related to this factor:

1. I am able to use ICT to better understand the subject content.
2. I am able to identify the subject topics that can be better presented with ICT.

(iii.) *Curriculum Design:* High scores for this factor indicate that the medical educator has gained skills such as planning a curriculum integrated with ICT, using ICT designs and teaching strategies integrated with ICT. The following are sample items related to this factor:

1. I am able to evaluate the factors that influence the planning of an ICT-infused curriculum.
2. I use appropriate ICT representations to present instructional content.

(iv.) *Practical Teaching:* High scores for this factor indicate that the medical educator has skills such as using ICT in instructional management and facilitating the achievement of the students. The following are sample items related to this factor:

1. I am able to indicate the advantages and disadvantages of ICT for instructional management.
2. I am able to use ICT to facilitate the achievement of teaching objectives.

(v.) *Assessment:* High scores for this factor indicate that the medical educator has gained skills such as using ICT technologies to assess student learning. The following are sample items related to this factor:

1. I know the types of technology-infused assessment approaches.
2. I am able to use ICT to assess students’ learning progress.

#### Attitude towards technology scale

Attitude towards Technology Scale (ATT), which was developed by Yavuz [21] to determine educators and educator candidates’ attitudes towards technology, consists of 5 factors and 19 items expressed a five-point Likert scale, as ‘definitely agree’ (5), ‘agree’ (4), ‘indifferent’ (3), ‘disagree’ (2) and ‘definitely disagree’ (1). The factors that form the scale are as follows: (*i*) not using technological tools in the education area, (*ii*) using technological tools in education area, (*iii*) the effects of technology on education, (*iv*) knowing how to use technological tools and (*v*) the evaluation of technological tools. The scale consists of 13 positive and 6 negative items. The reliability coefficient of the measurement tool was calculated using the Cronbach Alpha method and the reliability coefficient was found to be 0.87. The correlations of 19 items were calculated to reveal item discrimination and item difficulty were found to vary between .24–.68 [20]. Accordingly, the obtained data were coded by assigning a value from 5 to 1 to positive items, and a value from 1 to 5 to negative items.

The content of the scale factors can be described as follows:

(i.) *Not Using Technological Tools in Education* (5 items): Educators who get high scores from this factor possess negative attitudes towards technology, such as the use of technological tools in education is unnecessary, they are not economical in terms of time and they don’t have any impact on the motivation of the students. A sample of the items belonging to this factor are as follows:

1. Using the Internet in the learning process is a waste of time.
2. Using technological tools does not affect students’ motivation.

(ii.) *Using Technological Tools in Education* (4 items): Educators who achieved high scores from this factor believe that the use of technological tools in education is important and necessary and it will contribute to the gains of education. A sample of the items belonging to this factor are as follows:

1. Students should receive basic education on computer literacy.
2. Technological tools could be used for practice or revision.

(iii.) *The Effects of Technology on Education Life* (4 items): Educators who get high scores from this factor believe that technology is important and indispensable in education; its necessity is not limited to the classroom, it goes outside the school as well. A sample of the items belonging to this factor are as follows:

1. One does not have to use technological facilities in order to be successful in life.
2. Technological facilities have a positive effect on productive studying and learning.

(iv.) *Teaching How to Use Technological Tools* (4 items): Educators who achieved high scores from this factor believe that training featuring the use of technology should be provided within the educator training system and students should be informed about their capabilities. A sample of the items belonging to this factor are as follows:

1. Students should get advanced information on the usage of new technologies.
2. The use of new technologies in educator’s education should be increased.

(v.) *Evaluating technological tools* (2 items): Educators who get high scores from this factor have positive attitudes toward the effectiveness of technological tools. A sample of the items belonging to this factor are as follows:

1. Technological tools can only succeed when they address all the senses.
2. In order to be able to graduate from the university, the ability to “use the technological materials of the field” should be rated.

### Procedure

In the study, data were obtained by applying the scales to the participants. Participants answered demographic questions first, than they marked their level of agreement to the items on the scale. Filling the scale was optional and permission was granted from the school administrators and medical educators prior to the application of the scale. In order to determine the differentiation of the medical educators’ technology integration according to technological pedagogical content knowledge, the *Ward’s minimum variance hierarchical cluster analysis* was performed; *discriminant function analysis* was conducted to ensure the validity of the clusters; *one sample t-test* was conducted for the comparisons of the clusters to identify their characteristics and name them; *Levene* test and *ANOVA* was applied for the comparisons between clusters; and *post-hoc* analysis was conducted using the *Scheffe test* to identify the source of significant differences in the main affect. In addition, *Correlation analysis* was performed to determine the relationships between medical educators’ technology integration and their attitudes towards technology. The *chi-square independence test* was used to test the hypothesis about the clusters based on medical educators’ TPACK-Practical skills and the distribution of the medical educators according to implementation status of simulation-based medical education, department, and gender variables. In addition, the prediction power of the variables, such as gender, medical educators’ seniority, their attitudes towards technology, implementation of simulation-based medical education’s in their school and the department where they were working, and the assignment of the medical educators to one of the clusters formed by the clustering analysis was analyzed using the *Multinomial Logistic Regression*. The *forward stepwise* method was used in *Multinomial Logistic Regression.* Since both main and common affects were included in the hypothesis, very big parameter predictions and standard errors were obtained, and therefore it was decided that there existed *multicollinearity* among variables. In order to analyze multicollinearity, *Pearson correlation coefficients* of the continuous variables were calculated. The correlation between age and seniority was very high, thus the age variable was excluded from the model to ensure that the *Multinomial Logistic Regression* analysis explained abnormal behaviors (*r* = .89, *p <* .01). In order to check if there were other variables to be excluded, *likelihood ratio statistics* was performed. To determine the size of the dependent variable’s variance, *Cox and Snell R2* and *Nagelkerke R*^*2*^ values were checked.

## Results

In the study, TPACK-Practical skills were taken as the clustering variable while determining the differentiation of medical educators’ technology integration. In the conducted *Ward’s minimum variance hierarchical cluster analysis*, the graphical illustration of the data was examined as a *dendrogram* in order to determine the appropriate number of clusters and the examination of the dendrogram revealed several solutions consisting of three, four five clusters. Each clustering solution was evaluated and clusters belonging to these potential solutions were formed separately in order to find the most meaningful clustering solution. Accordingly, the solution with three clusters was chosen and the distribution of the clusters is presented in Table 1.

**Table 1:** Distribution of the Cluster Analysis Results about TPACK-Practical skills

Clusters	*n*	*%*
*Cluster* 1	116	38.5
*Cluster* 2	97	32.2
*Cluster* 3	88	29.2
Total	301	100

**Fig. 2:**
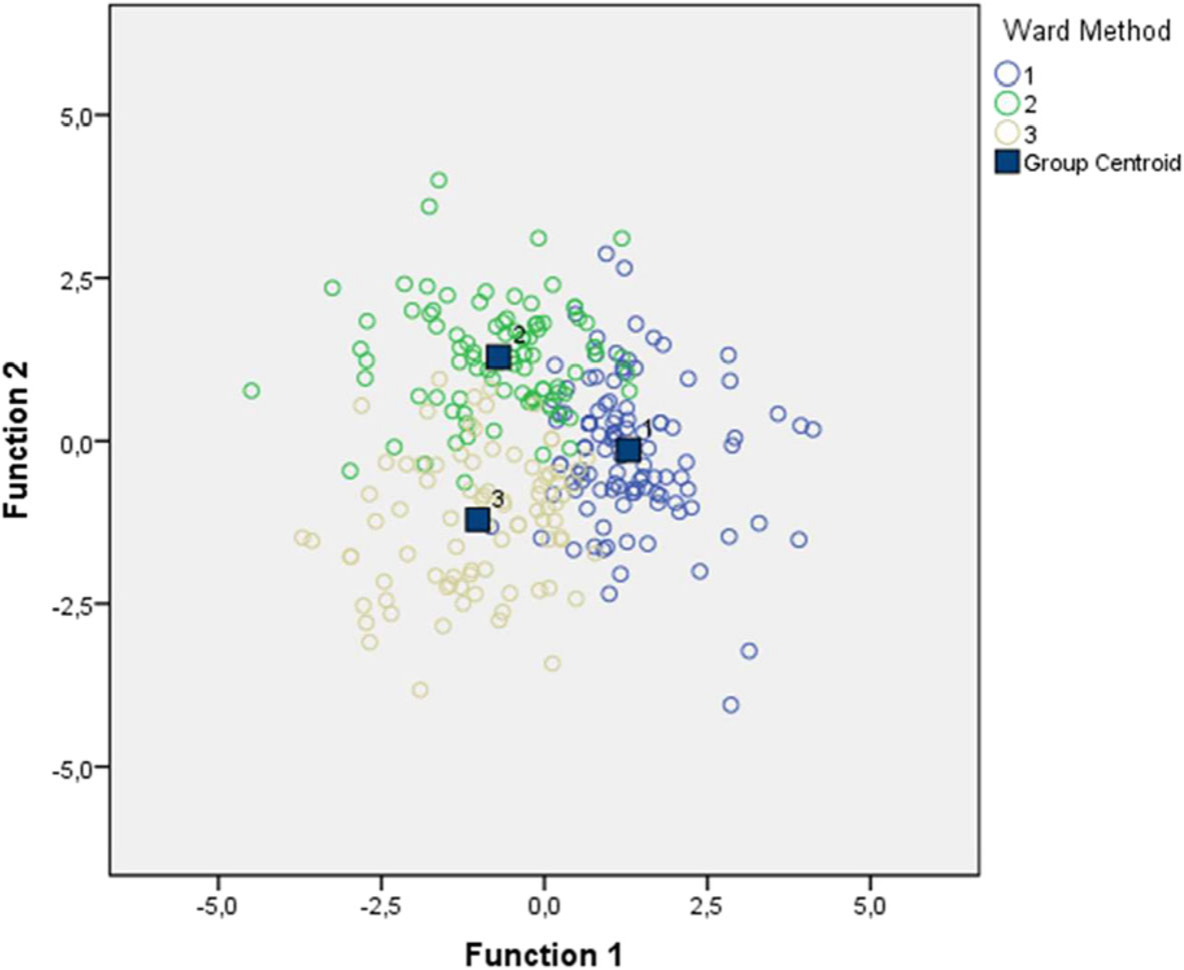
Canonical Discriminant Functions

The review of Table 1 shows that the number of medical educators in the clusters is 116, 97 and 88, respectively. To ensure the validity of the clusters obtained via clustering analysis, *discriminant function analysis* was conducted to the whole sample [22]. The results of this analysis are presented in Table 3. It can been seen that the three-cluster solution predicted 83*%* of cluster membership (See Table 2 and Fig. 2), whereas the four-cluster solution predicted 72*%* and the five-cluster solution predicted 65*%* of it. Consequently, the three-cluster solution was found to be the best solution for identifying similarities within medical educators’ groups and differentiations among groups.

**Table 2:** Cluster Membership Predicted by the Classification Matrix

Ward Method		Predicted Group Membership			Total
		*Cluster* 1	*Cluster* 2	*Cluster* 3	
Original Count	*Cluster* 1	108	5	3	116
	*Cluster* 2	10	81	6	97
	*Cluster* 3	11	8	69	88

83.9*%* of original grouped cases correctly classified

### Comparison of the clusters and determination of cluster characteristics

After the identification of the clusters, their characteristics were revealed and they were named accordingly. In addition, comparisons within and between clusters were reported.

#### Comparisons between clusters

In order to determine the characteristics of the clusters and to name them, the assumption of homogeneity of variance was checked (Levene test, *p* = .09; *p* = .24; *p* = .21; *p* = .69 and *p* = .62). Accordingly, it was decided that it was appropriate to perform an ANOVA test on the data (*p >* .05). As a result of ANOVA, it was found that there were significant differences between the mean scores achieved by the clusters in the following areas of TPACK-Practical scale: *learners* [*F*_(2, 298)_ = 19.41; *p <* .01], *subject content* [*F*_(2, 298)_ = 20.07; *p <* .01], *practical teaching* [F_(2, 298)_ = 59.42; *p <* .01] and *assessment* [F_(2, 298)_ = 5.98; *p <* .01]. There were no significant differences between the three clusters in terms of *curriculum design* [F_(2, 298)_ = 0.99; *p >* .05].

**Fig. 3:**
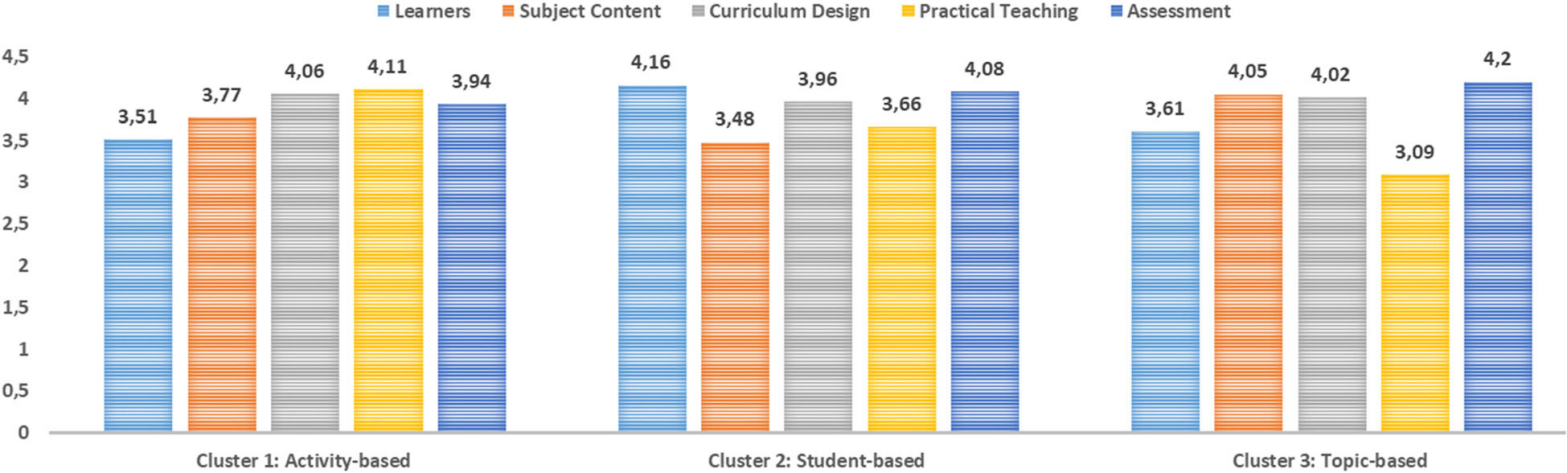
Mean Scores of the Clusters in Various Areas of the TPACK-Practical Scale

Regarding the direction of the differences between clusters, the difference was in favor of the second cluster (student-based) in the *learner’s* area. In the *subject content* area, the means of the first (activity-based) and third (topic-based) clusters were found to be higher than second cluster (student-based). In the *practical teaching* area, it was found that the mean of the first cluster (activity-based) was higher than the other two clusters. In the *assessments* area the difference was not based on one cluster; it was in favor of the second (student-based) and third (topic-based) clusters. As can be seen, the clusters, which possess advantages in some areas, were named after the names that form the conceptual basis of this area. The characteristics of these clusters and the graphic showing their mean scores in each area are displayed in Fig. 3.

The review of Fig. 3 shows that the highest mean of the activity-based cluster is on the *practical teaching* area (*M* = 4.11), whereas the lowest mean is on the *learners* area (*M* = 3.51). On the other hand, the highest mean of student-based cluster is on the *learners* area (*M* = 4.16), whereas the lowest mean is on the *subject content* area (*M* = 3.48). Regarding topic-based cluster, the highest mean is observed on the *assessment* area (*M* = 4.20), whereas the lowest mean is on the *practical teaching* area (*M* = 3.09). As can be seen from Fig. 3, the characteristics of the clusters are in line with their names.

#### Comparisons within clusters

After the identification of the TPACK-Practical Scale clusters’ characteristics and calculating the mean score in each area, comparisons were done within each cluster. In the study, the shape of TPACK-Practical skills among medical educators and the attitudes of the clusters, which consist of medical educators having similar skills, towards technology were especially considered. According to the hypothesis that Practical skills of the medical educators would vary according to their attitudes towards the source of TPACK-Practical skills, an individual-centered approach was adopted. In this context, the null hypothesis was tested.

The review of Table 3 revealed that there are positively correlated, negatively correlated and non-correlated factors among TPACK-Practical and ATT scales factors. The significant relationship between TPACK-Practical and ATT scales factors supports the hypothesis that Practical skills of the medical educators vary according to their attitudes towards the source of TPACK-Practical skills. Accordingly, it can be concluded that medical educators’ attitudes towards technology affect their TPACK-Practical skills.

**Table 3:** Correlation Matrix among the Factors of TPACK-Practical and ATT Scales’ Factors

Variables		1	2	3	4	5	6	7	8	9	10	11	12	13
TPACK-Practical Scale														
1	Using ICT to understand students	1												
2	Using ICT to understand subject content	.21^*^	1											
3	Planning ICT-infused curriculum	.23^**^	.29^**^	1										
4	Using ICT representations	.39^**^	.25^**^	.30^**^	1									
5	Using ICT-integrated teaching strategies	.29^**^	.42^**^	.29^**^	.52^**^	1								
6	Applying ICT to instructional management	.22^**^	.23^**^	.29^**^	.28^**^	.41^**^	1							
7	Infusing ICT into teaching contexts	.28^**^	.21^**^	.35^**^	.36^**^	.35^**^	.59^**^	1						
8	Using ICT to assess students	.37^**^	.18^**^	.30^**^	.41^**^	.40^**^	.14^*^	.19^**^	1					
Attitude Towards Technology Scale														
9	Not using technological tools in education	−.07	−.20^*^	−.01	−.01	−.22*^*^	−.01	−.02	−.01	1				
10	Using technological tools in education	.19^*^	.19^*^	−.05	.26^**^	.14^**^	.28^**^	.03	.04	−.35^**^	1			
11	The effects of technology on education life	.21^**^	.03	.07	.13^*^	.02	.17^*^	.21^**^	.01	−.34^**^	.74^**^	1		
12	Teaching how to use technological tools	.19^*^	.19^**^	.03	.18^*^	.42^**^	.26^**^	30^**^	.11^*^	−.29^**^	.65^**^	.72^**^	1	
13	Evaluating technological tools	.07	.05	.06	.19^*^	.21^**^	.06	.02	26^**^	−.26^**^	.68^**^	.69^**^	.63^**^	1

*N* = 301; *p < 0.05; **p < 0.01

H_2_ hypothesis -“The clusters formed according to TPACK-Practical skills of the medical educators and the distribution of the medical educators who were working in the schools where simulation-based medical education was implemented are independent”- which was formed to reveal the factors effecting medical educators’ technology integration process, was tested using the data obtained from the TPACK-Practical scale and demographic information.

The data about the clusters and medical educators who were working in the schools where simulation-based medical education was implemented and not implemented and the results of the chi-square independence test are presented in Table 4. According to Table 4, 82 medical educators (70.7*%*) from the *activity-based* clusters, 34 medical educators (35.1*%*) from the *student-based* clusters and 37 medical educators (42.0*%*) from the *topic-based* clusters were working in the schools where simulation-based medical education was implemented. According to these results, the hypothesis -“The clusters formed according to TPACK-Practical skills of the medical educators and the distribution of the medical educators working in the schools where simulation-based medical education was implemented are independent”- was rejected (*X*^2^_(2)_ = 36.41; *p* < .01). Consequently, it can be expressed that the implementation of simulation-based medical education affects the medical educators’ cluster.

**Table 4:** The Results of Chi-square Independence Test for the Implementation Status of Simulation-Based Medical Education (SBME) and Medical Educators’ Clusters

			SBME		Total
			Not in use	In use	
Cluster	Activity-based	*N*	34	82	116
		In cluster *%*	29.3	70.7	100
		SBME *%*	23.0	53.6	38.5
	Student-based	*N*	63	34	97
		In cluster *%*	64.9	35.1	100
		SBME *%*	42.6	22.2	32.2
	Topic-based	*N*	51	37	88
		In cluster *%*	58.0	42.0	100
		SBME *%*	34.5	24.2	29.2
Total		*N*	148	153	301
		In cluster *%*	49.2	50.8	100
		SBME *%*	100	100	100

*p* < .01, *X*^2^ = 36.41, *DF* = 2

H_3_ hypothesis -“The clusters formed according to TPACK-Practical skills of the medical educators and the department where they were working are independent”- was tested using the data obtained from the TPACK-Practical scale and demographic information. The results are displayed in Table 5.

**Table 5:** The Results of Chi-square Independence Test for the Department and Medical Educators’ Clusters

			Department			Total
			Basic sciences division	Medical sciences division	Surgical sciences division	
Cluster	Activity-based	*N*	45	40	31	116
		In cluster *%*	38.8	34.5	26.7	100
		Department *%*	43.7	42.6	29.8	38.5
	Student-based	*N*	39	24	34	97
		In cluster *%*	40.2	24.7	35.1	100
		Department *%*	37.9	25.5	32.7	32.2
	Topic-based	*N*	19	30	39	88
		In cluster *%*	21.6	34.1	44.3	100
		Department *%*	18.4	31.9	37.5	29.2
Total		*N*	103	94	104	301
		Between clusters *%*	34.2	31.2	34.6	100
		Department *%*	100,0	100.0	100.0	100.0

*p* < .01; *X*^2^ = 81.41; *DF* = 4

According to Tables 5, 45 of 116 medical educators (38.8*%*) from the *activity-based* cluster were working in the basic sciences division, 40 medical educators (34.5*%*) in the medical sciences division and 31 medical educators (26.7*%*) in the surgical sciences division. Besides, 39 medical educators (40.2*%*) from *student-based* cluster were working in the basic sciences division, 34 medical educators (35.1*%*) in the surgical sciences division and 24 medical educators (24.7*%*) in the medical sciences division. Regarding the *topic-based* cluster, 39 medical educators (44.3*%*) were working in the surgical sciences division, 30 medical educators (34.1*%*) in the medical sciences division and 19 medical educators (21.6*%*) in the basic sciences division.

According to these results, the hypothesis -“The clusters formed according to TPACK-Practical skills of the medical educators and the department where they were working are independent”- was rejected (*X*^2^_(4)_ = 81.41; *p* < .01). Consequently, it can be expressed that the department affect the medical educators’ cluster.

H_4_ hypothesis -“The clusters formed according to TPACK-Practical skills of the medical educators and their gender distribution are independent”- was tested using the data obtained from the TPACK-Practical scale and demographic information. The results are displayed in Table 6.

**Table 6:** The Results of Chi-square Independence Test for Gender and Medical Educators Clusters

			Gender		Total
			Male	Female	
Cluster	Activity-based	*N*	51	65	116
		In cluster *%*	44.0	56.0	100
		Gender *%*	44.3	34.9	38.5
	Student-based	*N*	35	62	97
		In cluster *%*	36.1	63.9	100
		Gender *%*	30.4	33.3	32.2
	Topic- based	*N*	29	59	88
		In cluster *%*	33.0	67.0	100
		Gender *%*	25.2	31.7	29.2
Total		*N*	115	186	301
		Between clusters *%*	38.2	61.8	100
		Gender *%*	100	100	100

*p* > .05; *X*^2^ = 1.99; *DF* = 2

According to the results, 65 medical educators (56.0*%*) from the *activity-based* clusters, 62 medical educators (63.9*%*) from the *student-based* clusters and 59 medical educators (67.0*%*) from the *topic-based* clusters were female. According to these results, the hypothesis -“The clusters formed according to TPACK-Practical skills of the medical educators and their gender distribution are independent”- was accepted (*X*^2^_(2)_ = 1.99; *p* > .05). As a result, it can be said that the gender of the medical educators’ doesn’t affect their cluster.

### Cluster definitions

The three clusters formed via the cluster analysis were compared within themselves and with other clusters in terms of both TPACK-Practical and ATT factors. The outcomes are discussed below.

#### Activity-based cluster

The activity-based cluster is a big cluster consisting of 116 (38*%*) medical educators. They got higher scores from practical teaching, especially from skills such as “*Using ICT-integrated teaching strategies*” and “*Using ICT on education management”*. The main characteristic, which differentiated this cluster from the others, is the even distribution of the mean scores from the five areas, compared to the other clusters.

Medical educators of the activity-based cluster have higher scores from the following factors compared to the other clusters: “*Use of Technological Tools in Education*”, “*Impact of Technology on Education*”, “*Knowing How to Use Technological Tools*” and “*Evaluation of Technological Tools*”. The majority of these medical educators (*%*70.7) work at schools where simulation-based medical education was implemented. 58*%* of the medical educators working in the school where the project was implemented fell in to this cluster. Medical educators mostly work at the basic sciences division (34*%*), and they form 47*%* of the medical educators working at the basic sciences division. The cluster has a balanced distribution in terms of gender.

#### Student-based cluster

Medical educators falling to in this cluster form approximately 32*%* of the participants (97 medical educators). They got higher scores from the factor “*Using ICT in understanding the learners”*. They have lower scores for “*Using ICT to understand subject content”* compared to the other clusters. However, these medical educators have high mean scores on the skills about running the technology in the assessment process. Their skill for using the technology in the teaching process is lower than the *activity-based* cluster. Their attitude towards technology is high. Compared to the topic-based cluster, they are aware of the impact of technology on education and they know well how to use technological tools. 35*%* of the medical educators working in the schools where simulation-based medical education was implemented fell in to this cluster. In other words, 64*%* of these medical educators work in schools where simulation-based medical education has not been implemented. In addition, medical educators of these clusters form half of the medical educators working at the basic sciences division. Regarding gender, approximately 59*%* are female.

#### Topic-based cluster

The topic-based cluster is the smallest cluster, consisting of 88 medical educators (29*%*). Medical educators of this cluster give importance to technology usage in the topic area. In terms of teaching the content, they are better than the *activity-based* and *student-based* groups. They have the “*Ability to use ICTs to better understand the subject matter content*” and the “*Ability to identify topic’s themes that could be presented in a better way with ICT*”. They know well the appropriate technology to be used for the topics. The attitude towards technology of these medical educators who select the technology according to topic is lower than in the other clusters. Even though a small proportion of these medical educators work in the schools where simulation-based medical education was implemented, the majority of basic and medical sciences divisions, simulation-based medical education has not been implemented. These divisions are comprised mostly of women (64.7%).

The research addressed the question -“What are the factors affecting the assignment of the medical educators to the clusters that were formed according to TPACK-Practical skills?”- The proposed model tested the H_5_ hypothesis; −“Medical educators’ seniority, attitude towards technology, simulation-based medical education’s implementation status in their school, department and gender variables affect their presence in the clusters formed according to TPACK-Practical skills”-. The predictor variables of the model generated in the study were seniority, ATTS scores, implementation status of simulation-based medical education, department and gender. The outcome variable of the analysis is being assigned to a cluster. The prediction powers of seniority, attitude towards technology, implementation status of simulation-based medical education at their school, department where they work and gender variables were analyzed via *Multinomial Logistic Regression*. In order to ensure that *Multinomial Logistic Regression* analysis explains abnormal behaviors, the age variable was not used in the analysis as a predictor variable (See Methodology).

In Table 7, the *-2log likelihood* fit index shows the fit of the estimated model. This value was found to be 599.1 at the beginning and 458.2 at the end. According to Hair, Balack, Babin, Anderson, and Tatham [23], the difference between the initial and final values of likelihood should be evaluated for the interpretation of -2log likelihood difference. According to Table 7, this difference is significant (*X*^2^_(20)_ = 140.9; *p* < .01). Accordingly, it was concluded that the predictor variables make a significant contribution to improve the fit of the estimated model.

**Table 7:** Model Likelihood Values

	−2 Log Likelihood	Chi-Square	DF	*p*
Intercept only	599.1	140.9	20	.00
Final	458.2			

Table 8 presents the results of goodness of fit test. According to Garson [24], the goodness of fit test evaluates the fit of logistic regression model as a whole. The significance of this test results (*p >* .05) shows that the model-data fit is at an appropriate level. According to Table 8, the model-data fit of the model is sufficient (*X*^2^_(573)_ = 539.5; *p* > 05).

**Table 8:** Results of Goodness of Fit Test

Goodness of Fit			
	Chi-Square	DF	*p*
Pearson	539.5	573	.18
Deviance	417.3	573	.91

Corrected *R*^*2*^ values are shown in Table 9. Higher values mean better fit [25]. As can be seen from Table 9, this value is .79, which means that the model developed using dependent variables explains the independent variable with 79*%* variance estimation.

**Table 9:** Corrected R^2^ Values

Corrected *R*^2^	
Cox and Snell	.79
Nagelkerke	.88

The results of the *Multinomial Logistic Regression* are presented in Table 10. The reference cluster of the *Multinomial Logistic Regression* analysis is the *activity-based* cluster. According to the results of the Wald test presented in Table 10, it was found that “*Teaching how to use technological tools*”, which is the fourth factor of the ATT scale, decreases the likelihood of being in the *student-based* cluster compared to being in *activity-based* cluster, which is the reference cluster. One unit increase in the “*Teaching how to use technological tools*” factor, causes a 71*%* decrease [(0.29–1)× 100] on the odds of being in the *student-based* cluster. In other words, it means that the “*Teaching how to use technological tools*” factor increases the likelihood of being in *activity-based* cluster 3.44 times (OR = 1/0.29 = 3.44), compared to being in the *student-based* cluster.

**Table 10:** Results of the Multinomial Logistic Regression

Ward Method		B	Std. Error	Wald	DF	*p*	Exp(*β*)
Student-based	Intercept	−3.11	1.11	7.98	1	.00	
	Seniority	.03	.03	1.78	1	.21	.92
	ATT1	−.23	.19	1.09	1	.33	.81
	ATT2	.39	.31	2.35	1	.15	1.45
	ATT3	.58	.41	2.89	1	.09	1.93
	**ATT4**	**−1.12**	**.38**	**8.78**	**1**	**.00**	**.29**
	TTF5	.39	.27	1.86	1	.22	1.42
	[**SBME = not in use**]	**1.49**	**.59**	**5.79**	**1**	**.00**	**4.38**
	[SBME = in use]	0^b^	–	–	0	–	–
	[**Basic sciences division**]	**2.70**	**.69**	**13.96**	**1**	**.00**	**17.01**
	[Medical sciences division]	−.61	.64	.77	1	.41	.49
	[Surgical sciences division]	0^b^	–	–	0	–	–
	[Female]	.25	.35	.51	1	.53	1.31
	[Male]	0^b^	–	–	0	–	–
Topic-based	Intercept	−3.29	1.21	8.29	1	.00	
	Seniority	.04	.04	.87	1	.41	.91
	ATT1	−.37	.25	3.36	1	.09	.71
	**ATT2**	**.58**	**.31**	**4.38**	**1**	**.01**	**1.91**
	ATT3	.38	.35	.77	1	.41	1.53
	**ATT4**	**−1.28**	**.37**	**12.12**	**1**	**.00**	**.39**
	**ATT5**	**.71**	**.34**	**5.32**	**1**	**.00**	**2.38**
	[SBME = not in use]	.65	.61	1.17	1	.32	2.01
	[SBME = in use]	0^b^	–	–	0	–	–
	[**Basic sciences division**]	**3.88**	**.75**	**28.86**	**1**	**.00**	**4.87**
	[Medical sciences division]	.79	.65	1.64	1	.26	2.17
	[Surgical sciences division]	0^b^	–	–	0	–	–
	[Female]	.48	.39	1.74	1	.21	1.43
	[Male]	0^b^	–	–	0	–	–

According to the Wald test, the lack of implementation of simulation-based medical education in medical educators’ schools increases the likelihood of being in the *student-based* cluster 4.3 times (OR = 4.38). This finding can be expressed as follows: the implementation of simulation-based medical education increases the likelihood of being in the *activity-based* cluster 4.38 times. In addition, working in primary schools increases the likelihood of being in the *student-based* cluster (compared to *activity-based* cluster) 17.01 times (OR = 17.01).

According to the Wald test, *“Using technological tools in education”* and “*Teaching how to use technological tools*”, which are the second and fourth factors of the ATT scale, have an increasing effect of being in the *topic-based* cluster compared to the reference cluster by approximately 1.91 (OR = 1.91) and 2.38 (OR = 2.38) times, whereas “*Evaluating technological tools*”, which is the fifth factor, leads to a 61.1*%* decrease [(0.39–1)× 100] on the odds of being in the *topic-based* cluster.

Regarding the variable featuring the workplace of the medical educators, working in the basic sciences division increases the likelihood of being in the *topic-based* cluster by 4.87 times (OR = 4.87) compared to being in the *activity-based* cluster.

## Discussion

### Clusters formed based on the TPACK-practical skills of the medical educators and their characteristics and discussion

It was found that, in terms of technology integration medical educators were grouped under 3 clusters and the developed model predicted 83*%* of the cluster membership. In addition it was found that these clusters were not correlated with gender, but significant relationships were identified between the cluster of the medical educators and their school type and the implementation status of simulation-based medical education in their school.

The review of the clusters and their characteristics showed that there are similarities between the items that are used in the design stage of the education programs and the relationships of these items among them. Learner centered approaches are based on the assumption that students are located at the center of the program. In these designs, teaching focuses mostly on the learner, rather than the program, learning or administrative body. Individuals and their identities are crucial [26]. According to Ornstein and Hunkings [27], in this design students need the methods and materials used in different subject areas while solving the problems that they encounter. The review of the student-based cluster’s characteristics obtained from the research shows that medical educators achieved high scores on the “U*sing ICT to understand students*” factor. Accordingly, it can be said that medical educators who belong to the cluster that places students at the center of teaching (such as learning disability, individual differences, understanding the students), learner centered program design, implementation process and assessment.

Considering the characteristics of the students-based cluster, they are mainly composed of medical educators working in basic sciences divisions. In addition, this cluster was dominated by female medical educators. Regarding topic-centered approaches, schools following this design have a strong academic rationalism. The effectiveness of the textbooks and medical educator training are important. In the design of the subject area, which is one of the topic-centered designs, the underlining idea is that topics are held the best in the textbooks. In topic design the content is emphasized, and students’ interests, needs and lives are put on the back burner [27]. According to the results of the study, the topic-based cluster achieved a higher mean on the use of technology for the subject area, compared to other clusters. Regarding the characteristics of the activity-based cluster, it can be seen that these medical educators understood the importance of technological tools and equipment on the technology integration process and they possess adequate material about how to run technology in education.

The characteristics of the clusters discussed above within the context of TPACK-Practical, can be explained according to the approach suggested by Mandinach and Cline [28], which was proposed within the context of ICT usage stages in the process of technological integration and which considered individual differences on the dissemination of the innovations in schools. This approach argues that individual characteristics are important in the dissemination of educational technology in schools and the individual may be at one of four different stages. These stages are expressed as survival, mastery, impact and innovation. At the survival stage, medical educators attempt to use technology by trial and error, while maintaining their positions in the classrooms. At the mastery stage, their technical abilities increase, new forms of interaction appear, new learning strategies and different curriculum models are developed, and consequently they become less dependent on field specialists. At the impact stage, classrooms become more student centered, and thus the variety of technological learning activities and the use system applications increase. And finally when the innovation stage is reached, medical educators restructure the curriculum and learning activities, and carry the operations and the content to a more advanced level [28]. This approach, which can explain topic-based and student-based clusters, is not very descriptive for activity-based medical educators who were mostly working at the schools where simulation-based medical education was implemented. The reason for this may be the external direct support (in-service training, software, technical information, educational design, etc.) provided at survival and mastery stages and the influence of the factors outside of school. According to Jen, Yeh, Hsu, Wu and Chen [29], TPACK-Practical is developed based on experiences of teaching with technology and instructional reflection, and neither academic nor application TPACK-Practical can effectively be enhanced when technology are not implemented or experimented within classrooms.

### Results about the factors affecting the assignment of the medical educators to the clusters formed according to TPACK-practical skills and discussion

In the study, the factors, which affect the assignment of the medical educators to the clusters formed according to TPACK-Practical skill, were examined, and it was found that “*Teaching how to use technological tools*” and the implementation of simulation-based medical education increases the likelihood of being in the activity-based cluster, compared to being in the student-based cluster. This fact can be explained as the positive affect of the technological infrastructure and the trainings provided after the implementation of simulation-based medical education. Having the ability of using technological tools enables medical educators to make important progress in fulfilling their shortcomings of technological knowledge. Researches shows that medical educators who acquired hi-level technology usage skills are more willing to use technology in their classrooms [30, 31]. Yavuz-Konokman, Yanpar-Yelken and Sancar-Tokmak [32] stated that TPACK perceptions of the medical educators differ according to the level of technology usage. Chai et al. [33] suggested that individuals who follow the development speed of ICT can integrate these technologies with pedagogical values more easily for teaching purposes. According to there was no relationship between how medical educators were thinking about technology integration and their self-assessment of technology integration competence. Accordingly, it can be said that simulation-based medical education and the training received in the way of gaining TPACK skills made an important contribution to the medical educators of the activity-based cluster in reaching the point of technology integration that they have arrived. Regarding the results of the research, it was found that working in primary schools increases the likelihood of being in student-based and topic-based clusters compared to being in the activity-based one. This fact is an indication that the technology integration process follows a different path in these schools, compared to activity-based cluster’s medical educators. It may be because the implementation of simulation-based medical educations varies according to department and it is different in the clusters as well. The evaluation of technological tools, which affect being in the topic-based cluster, shows that these medical educators put place an emphasis on the topic in of technology integration and they evaluate the tools in terms of the value of the gains for these topics.

## Conclusion

In this study, technology integration of the medical educators, arising from the integration of ICT in the learning-teaching process, was addressed via the TPACK-Practical model and it was found that medical educators were grouped under three clusters according to their technology integration level. Afterwards, the characteristics of these clusters were identified, the factors affecting integration process were revealed, how the resulting clusters were influenced by these factors and the reasons behind it were explained considering various variables in Turkey. The examination of the current status of medical educators’ technology integration, the identification of the conditions and the demonstration of the contradictions can be performed in the context of Activity Theory, which has a socio-cultural and historic perspective. Basic principles of activity theory and the items of the activity system may be the loadstar on the multidimensional study of the integration process. Activity Theory was shaped by the thought and consciousness studies of Vygotsky, and its basic interest is to understand the interactions between the individual, other humans and man-made objects [34, 35]. According to the principles of this theory, considering the conditions in which the actions occurred -along with the technology usage of the subject/medical educators and the identification of their goals- realizing these actions can be explained on the basis of the theory’s hierarchical structure. In addition, the process of technology integration was born from a need, which is often expressed as increasing the quality of the learning outcomes and the effectiveness of teaching, thus the initiation of ICT usage to satisfy various needs that can be described as educational, can be explained with the principle of object orientation. For example, activity, student and topic based clusters use technology for different purposes, the educational aims targeted by each school level are different and medical educators attempt to increase the effectiveness of the instruction for different purposes. In addition, the changes occurring in medical educators’ teaching approach, application of the process and purposes of using ICT during the integration activity, apparent and abstract changes in people caused by the technology, and the interaction of the changes occurred in affection and application dimensions are in line with the internalization and externalization principles of the theory. Medical educators’ attitude towards technology and the effects of this attitude on the sub-factors can be explained by the internalization principle, whereas the technological equipment that medical educators get with the implementation of simulation-based medical education, which affected their TPACK-Practical performance, can be explained by the externalization principle. The usage of ICT as a tool for realizing educational goals of the learning-teaching process is supported by the principle of mediation, whereas the acceptance of integration as a process, not as an event, and the requirement of long terms examinations is in line with the principle of development. According to Demiraslan and Koçak-Usluel [36], ICT integration and the TPACK process form a dynamic system. Therefore, it is important to reveal future trends via the examination of the past and present status of the process and make comments about the process by monitoring the changes.

## Directions for future research and limitations

The suggestions developed for TPACK research, medical educator training process technology integration and affective teaching, and future studies are as follows:

- Further studies featuring the factors that may affect TPACK factors, other than gender, department, seniority and attitude towards technology, can be conducted to fulfill the gap in the literature.
- Designing qualitative studies aiming to determine technology integration of the medical educators in terms of TPACK skills may reveal the role of the variables affecting integration processes.
- The provision of technological infrastructure and software required for the improvement of assessment skills, accompanied with an adequate training about the application.
- The position and importance of the technology should be explained to the medical educators and they should be convinced that the technology integration process is not limited to school life.
- Prior to in-service training, medical educators can be classified according to TPACK components; in-service training can be tailored according to the contribution of these components to TPACK.

It should be noted that the collection of the data by self-reporting might have caused subjectivity and distortion of the relationships between variables. The most important limitation of this study is the common method bias. The main reason of this limitation is the collection of the data from a single source (medical educators). This might have caused an artificial increase of the observed correlations. Although the aforementioned limitation cannot be fully eliminated from the study, the errors can be reduced to the minimum level. Therefore, the necessary precautions were taken during the data collection phase via the applications suggested at the beginning of the paper. First of all, the validity and reliability of the scales used in data collection stage were checked. Second, the participants were clearly informed that the responses would be kept confidential and they would not be revealed in any way. In addition the questionnaire is designed in a way that the scale items related to independent variables are listed before the ones related to dependent variables.

## Data Availability

All data generated or analyzed during this study are included in this manuscript. The raw or analyzed data of the current study are available on reasonable request.

## Abbreviations

ATTS: Attitude towards Technology Scale
ICT: Information and Communication Technology
PCK: Pedagogical Content Knowledge
TCK: Technological Content Knowledge
TPACK: Technological Pedagogical Content Knowledge
TPK: Technological Pedagogical Knowledge

## References

[1] Rogers E (2003). Diffusion of innovations.

[2] Abbitt J (2011). Measuring technological pedagogical content knowledge in preservice teacher education: a review of current methods and instruments. J Res Technol Educ.

[3] Doering A, Scharber C, Miller C, Veletsianos G (2009). GeoThentic: Designing and assessing with technological pedagogical content knowledge. Contemp Issues Technol Teach Educ.

[4] Graham C, Burgoyne N, Cantrell P, Smith L, Clair L, Harris R (2009). TPACK development in science teaching: measuring the TPACK confidence of inservice science teachers. TechTrends..

[5] Alinier G (2007). A typology of educationally focused medical simulation tools. Medical Teacher.

[6] Patrik J (2002). Simulation. Training: research and practice.

[7] Bradley P (2006). The history of simulation in medical education and possible future directions. Med Educ.

[8] McGaghie W, Issenberg S, Petrusa E (2010). Scalese. R. a critical review of simulation-based medical education research: 2003-2009. Med Educ.

[9] Kneebone R (2003). Simulation in surgical training: educational issues and practical implications. Med Educ.

[10] Weller J (2004). Simulation in undergradute medical education: bridging the gap between theory and practice. Med Educ.

[11] Yeh Y, Lin T, Hsu Y, Wu H, Hwang F (2015). Science teachers’ proficiency levels and patterns of TPACK in a practical context. J Sci Educ Technol.

[12] Koehler M, Mishra P (2005). What happens when teachers design educational technology? The development of technological pedagogical content knowledge. J Educ Comput Res.

[13] Angeli C, Valanides N (2009). Epistemological and methodological issues for the conceptualization, development, and assessment of ICT–TPCK: advances in technological pedagogical content knowledge (TPCK). Comput Educ.

[14] Koehler M, Mishra P (2009). What is technological pedagogical content knowledge. Contemp Issues Tech Teach Educ.

[15] Koh J, Chai C, Tsai C (2013). Examining practicing teachers’ perceptions of technological pedagogical content knowledge (TPACK) pathways: a structural equation modeling approach. Instr Sci.

[16] Van-Driel J, Verloop N, De-Vos W (1998). Developing science teachers’ pedagogical content knowledge. J Res Sci Teach.

[17] Yeh Y, HsU Y, Wu H, Chien S (2017). Exploring the structure of TPACK with video-embedded and discipline-focused assessments. Comput Educ.

[18] Jang S, Chen K (2010). From PCK to TPACK: developing a transformative model for pre- service science teachers. J Sci Educ Technol.

[19] Yeh Y, Hsu Y, Wu H, Hwang F, Lin T (2013). Developing and validating technological pedagogical content knowledge-practical (TPACK-practical) through the Delphi survey technique. Br J Educ Technol.

[20] Creswell J (2005). Educational research: planning, conducting, and evaluating quantitaive and qualitative research NY: Pearson.

[21] Yavuz S (2005). Developing a technology attitude scale for pre-service chemistry teachers. Turk Online J Educ Technol.

[22] Romensburg H (1984). Cluster analysis for researchers.

[23] Hair J, Black W, Babin B, Anderson R, Tatham R (2006). Multivariate data analysis upper.

[24] Garson G (2008). Discriminant function analysis.

[25] Field A (2005). Discovering statistics using SPSS for Windows.

[26] Dolance M (2003). The learner-centered curriculum model. A structured framework for technology planning. Center Appl Res.

[27] Ornstein A, Hunkins F (1998). Curriculum: foundations, principles, and issues.

[28] Mandinach E, Cline H (1994). Classroom dynamics: implementing a technology-based learning environment.

[29] Jen T, Yeh Y, Hsu Y, Wu H, Chen K (2016). Science teachers’ TPACK- practical: standard-setting using an evidence-based approach. Comput Educ.

[30] Hammond M, Fragkouli E, Suandi I, Crosson S, Ingram J, Johnston-Wilder P (2009). What happens as student teachers who made very good use of ICT during pre-service training enter their first year of teaching. Teach Dev.

[31] Paraskeva F, Bouta H, Papagianna A (2008). Individual characteristics and computer self-efficacy in secondary education teachers to integrate technology in educational practice. Comput Educ.

[32] Yavuz-Konokman G, Yanpar-Yelken T, Sancar-Tokmak H (2012). Sınıf öğretmeni adaylarının TPAB’lerine ilişkin algılarının çeşitli değişkenlere göre incelenmesi: Mersin üniversitesi örneği. Kastamonu Eğitim Fakültesi Dergisi.

[33] Chai C, Koh J, Tsai C, Tan L (2011). Modeling primary school pre-service teachers’ technological pedagogical content knowledge (TPACK) for meaningful learning with information and communication technology (ICT). Comput Educ.

[34] Nardi B (1996). Context and consciousness: activity theory and human computer ınteraction.

[35] Minnis M, Steiner V (2000). Are we ready for a single, ıntegrated theory?. Essay Review of Perspectives on Activity Theory.

[36] Demiraslan Y, Koçak-Usluel Y (2005). Bilgi ve iletişim teknolojilerinin öğrenme-öğretme sürecine entegrasyonunda öğretmenlerin durumu. Turkish Online J Educ Technol.

